# A three gene immunohistochemical panel serves as an adjunct to clinical staging of patients with head and neck cancer

**DOI:** 10.18632/oncotarget.18568

**Published:** 2017-06-19

**Authors:** Chin-Ann J. Ong, Nicholas B. Shannon, Stefan Mueller, Sze Min Lek, Xuan Qiu, Fui Teen Chong, Ke Li, Kelvin K.N. Koh, Gerald C.A. Tay, Thakshayeni Skanthakumar, Jacqueline S.G. Hwang, Tony Kiat Hon Lim, Mei Kim Ang, Daniel S.W. Tan, Ngian-Chye Tan, Hiang Khoon Tan, Khee Chee Soo, N. Gopalakrishna Iyer

**Affiliations:** ^1^ Department of General Surgery, Singapore General Hospital, S169856, Singapore; ^2^ Cancer and Stem Cell Biology, Duke-NUS Graduate Medical School, S169857, Singapore; ^3^ Division of Surgical Oncology, National Cancer Centre, S169610, Singapore; ^4^ Cancer Therapeutics Research Laboratory, National Cancer Centre, S169610, Singapore; ^5^ Department of Anatomical Pathology, Singapore General Hospital, S169856, Singapore; ^6^ Department of Medical Oncology, National Cancer Centre, S169610, Singapore; ^7^ Singhealth Duke-NUS Head and Neck Centre, Singhealth, S169856, Singapore

**Keywords:** head and neck squamous cell carcinoma, immunohistochemistry, genomics

## Abstract

**Background:**

Current management of head and neck squamous cell carcinoma (HNSCC) depends on tumor staging. Despite refinements in clinical staging algorithms, outcomes remain unchanged for the last two decades. In this study, we set out to identify a small, clinically applicable molecular panel to aid prognostication of patients with HNSCC.

**Materials and Methods:**

Data from The Cancer Genome Atlas (TCGA) was used to derive copy number aberrations and expression changes to identify putative prognostic genes. To account for cross entity relevance of the biomarkers, HNSCC (*n =* 276), breast (*n =* 808) and lung cancer (*n =* 282) datasets were used to identify robust and reproducible markers with prognostic potential. Validation was performed using immunohistochemistry (IHC) on tissue microarrays of an independent cohort of HNSCC (*n =* 333).

**Findings:**

Using GISTIC algorithm together with gene expression analysis, we identified six putative prognostic genes in at least two out of three cancers analyzed, of which four were successfully optimized for automated IHC. Of these, three were successfully validated; each molecular target being significantly prognostic on univariate analysis. Patients were differentially segregated into four prognostic groups based on the number of genes dysregulated (*p* < 0.001). The IHC panel remained an independent predictor of survival after adjusting for known survival covariates including clinical staging criteria in a multivariate Cox regression model (*p* < 0.001).

**Interpretation:**

We have identified and validated a clinically applicable IHC biomarker panel that is independently associated with overall survival. This panel is readily applicable, serving as a useful adjunct to current staging systems and provides novel targets for future therapeutic strategies.

## INTRODUCTION

Apart from prognostication, tumor staging in head and neck squamous cell carcinoma (HNSCC) plays a major role in treatment decisions. Patients with higher tumor stage benefit from the addition of adjuvant treatment which includes radiotherapy, chemotherapy (including cisplatin, 5-fluorouracil and/or taxol/taxanes) and biologic therapy (such as monoclonal antibodies against EGFR, e.g. cetuximab) [1–3]. Notwithstanding, overall outcomes have remained unchanged for the last two decades [4–8]. Undoubtedly, the poor prognosis can be attributed to the aggressive biological behavior of HNSCC. However, better patient stratification could also be an important determinant to identify patients expected to have poorer outcomes, hence treated more aggressively a priori.

Currently, staging for HNSCC is based on clinical and/or pathologic features: tumor size (T stage), presence or absence of nodal (N stage) and distant metastasis (M stage); forming the TNM classification. However, there is ample data to suggest that this system has its limitations, and several trials have already supported the addition of other adverse features to the staging algorithm, including margin status, depth of invasion, perineural invasion, lymph node density, and extra-capsular spread [9, 10]. Importantly, a number of these have important implications on management strategies, such as the addition of adjuvant chemotherapy [1, 11]. Even with the addition of a range of clinical data, there exists substantial variation within subgroups that are likely a reflection of the genetic and biologic make-up of the tumor itself.

Therefore, an ideal staging system for any tumor subtype should include clinical and molecular indicators that can portend the biological behavior of each individual tumor. Molecular prognostication is already an important component in HNSCC. The identification that a subset of oropharyngeal cancers is associated with the human papillomavirus (HPV), and that this has important prognostic and potentially therapeutic implications is a major paradigm shift [12]. In this situation, a simple immunohistochemistry (IHC) stain for p16 (as a surrogate marker), identifies what appears to be a distinct disease phenotype; a number of studies are underway exploring whether these should be treated differently from the usual protocols for HNSCC. The current experience with HPV and oropharyngeal cancer highlights several important issues in molecular prognostication. First, it is imperative that molecular prognostic tools should be used in concert with clinical staging algorithms rather than in isolation. The combination may result in refining or uncovering deficiencies in long-held views on tumor staging [13, 14]. Second, these markers need to be validated across large cohorts, using simple and reproducible assays such as IHC that are not biased in specific populations. Third, the applicability of these in therapeutic decisions require appropriate trials based on the biomarker itself, such as those currently underway for HPV-positive oropharyngeal cancer. Fourth, while not critical or essential, these markers could also be useful in studying tumor biology and molecular characteristics which in turn could direct rational application of targeted and personalized therapy [15]. To date, few markers meet these criteria; although several have been identified [16, 17], few have found their way into clinical practice [4, 18–21].

The objective of this study was to utilize publically-available expression and genomic data from The Cancer Genome Atlas (TCGA) to identify a suite of putative molecular prognostic markers, and validate these using IHC on tissue microarrays, in a completely different population cohort, in concert with a range of clinic-pathologic factors.

## RESULTS

### Identification of potential prognostic markers

Analysing the TCGA data, three amplicons (11q13, 3q22-29, 5p15) were associated with a number of genes for which expression data was prognostic in head and neck and in either breast or lung cancers (requiring *p <* 0·05). To generate the final list of targets to take forward for validation, we selected the two genes with the most significant *p*-value for survival association for each amplicon. The final targets selected were:

Anoctamin 1 (ANO 1) [11q13], Cortactin (CTTN) [11q13], Exocyst Complex Component 3 (EXOC 3) [5p15], Chaperonin Containing TCP1, Subunit 5 (CCT5) [5p15], Signal Sequence Receptor, Gamma (SSR3) [3q22-29] and ATPase Type 13A3 (ATP13A3) [3q22-29] The results for the prognostic significance of each target selected are shown in Supplementary Figure 1.

### Generation of the 3 gene prognostic biomarker panel

Of the six genes listed above, four were successfully optimized for immunohistochemistry (ANO1, EXOC3, SSR3 and ATP13A3), and these were subjected to validation on the TMAs. For internal validation of the dataset, survival based on AJCC stage was calculated (Supplementary Figure 2). Univariate Cox regression analysis showed that the IHC scores for each of the markers were correlated to OS. Dysregulation of ATP13A3, SSR3 and ANO1 showed a significantly increased HR of 1·45 (CI: 1·09 to 1·93; *p =* 0·01), 1·82 (CI: 1·2 to 2·78; *p <* 0·01) and 1·72 (CI: 1·21 to 2·46; *p <* 0·01) respectively (Supplementary Table 1). EXOC 3 showed an elevated HR of 1·12 (CI: 0·84 to 1·49) for OS, which however was not significant (*p =* 0·44). Hence, we only included ATP13A3, SSR3 and ANO1 in the further analysis. Supplementary Table 2 shows the number of dysregulated markers per AJCC stage.

### Validation of the 3 gene prognostic maker panel

**Figure 1:**
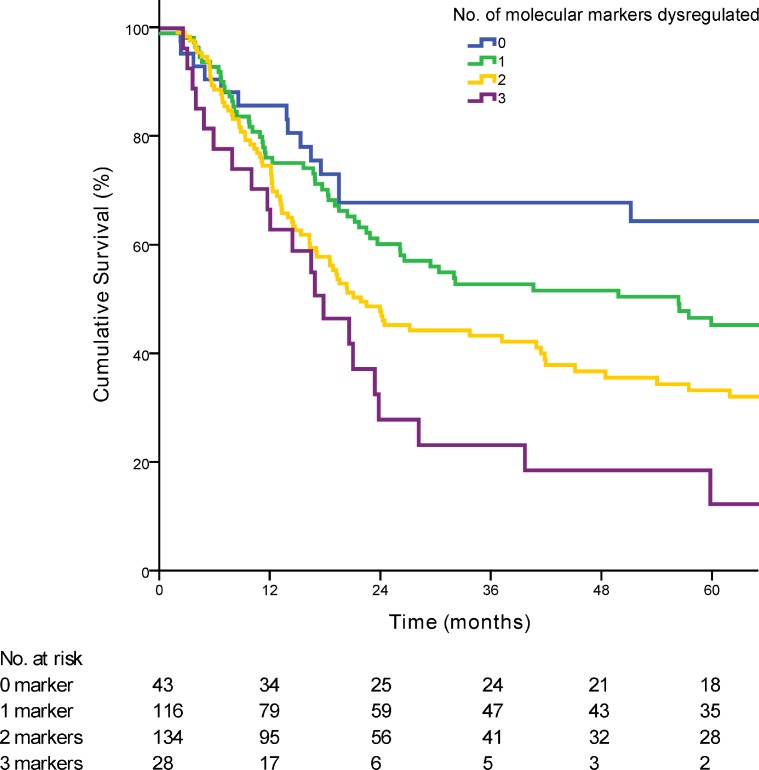
Kaplan-Meier curve demonstrating the survival of patients based on the number of dysregulated markers (*p <* 0·01) The number of patients at risk at each time point is displayed in the Table under the graph.

**Figure 2:**
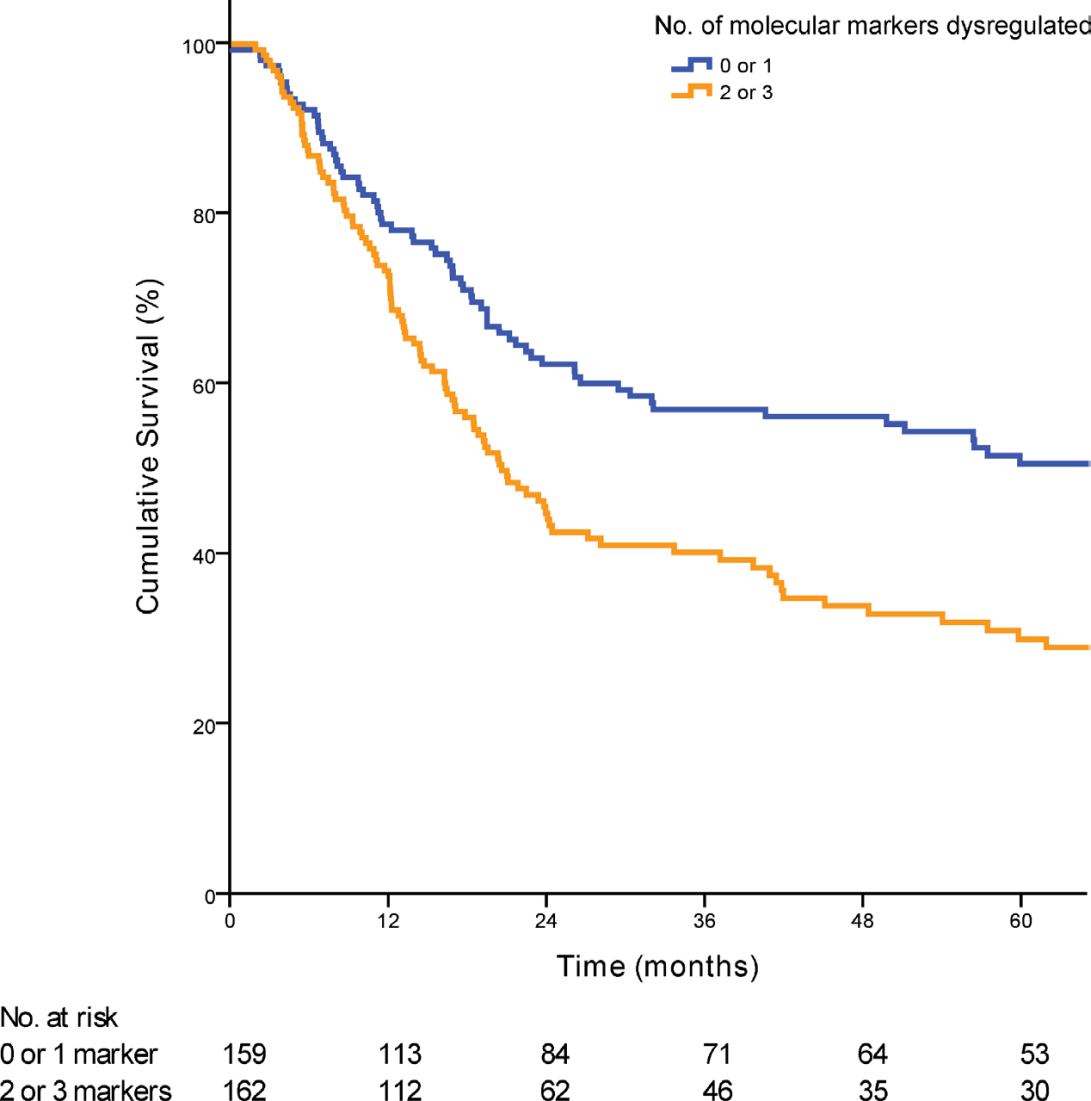
Kaplan-Meier curve demonstrating the prognostic significance of the prognostic biomarker panel when applied to the validation cohort (*p* < 0·01)

Dysregulation of 0, 1, 2 or 3 of the molecular markers showed a median survival of 60·00, 51·47, 23·35 and 18·85 months respectively (*p <* 0·01) (Figure 1). Each additional dysregulated molecular marker showed an increased HR by 1·47 (95% CI 1·23 to 1·80; *p <* 0·01). For clinical staging, patients were grouped into early stage (AJCC stage 1 and 2; *n =* 67) and late stage (AJCC stage 3 and 4; *n =* 266). When patients in the validation cohort were grouped according to the numbers of dysregulated markers, there was no statistical significant difference in the median OS between patients with 0 or 1 dysregulated marker (median OS 90·2 vs. 56·3 months; *p =* 0·16). Hence patients were further grouped according to either 0 or 1 marker dysregulated versus 2 and 3 markers dysregulated. Overall patients with 2 or 3 dysregulated markers showed a much poorer OS when compared with 0 or 1 dysregulated markers (median OS 66·2 vs 20·5 months; *p <* 0·01) (Figure 2). Post hoc power calculations of the prognostic effect of each individual and combined gene panel are detailed in Supplementary Table 3.

### Independent association of the three gene marker panel with OS

**Figure 3:**
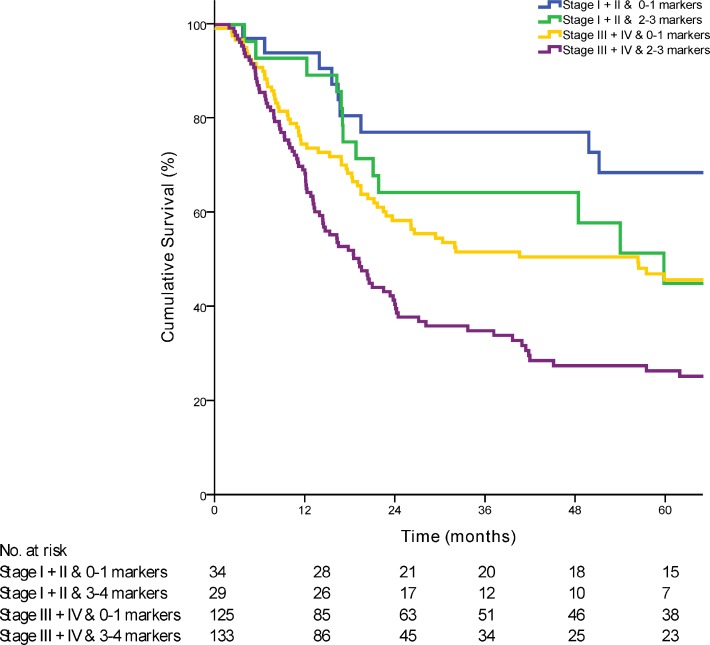
Kaplan-Meier curve comparing survival of patients based on the combination of AJCC stage with the prognostic marker panel (*p* < 0·01)

Having shown the significant impact of the three-gene marker panel on OS, a Cox multivariate regression analysis was performed to identify the independent association of the marker panel with OS when taking clinic-pathological features and known risk factors affecting outcome into account. We analyzed the prognostic value of the marker panel by performing a univariate and multivariate Cox regression analysis (Table 1). In the multivariate analysis the prognostic panel showed a HR of 2·23 (95% CI 1·41 to 3·54; *P <* 0·01). When combined with patients with early stage or late stage disease, patients with 2 or 3 genes dysregulated had a poorer prognosis compared to patients with 0 or 1 gene dysregulated (Figure 3).

**Table 1:** Univariate and multivariate survival analysis in the validation cohort

Characteristics	Univariate			Multivariate		
	HR	95% CI	*p*	HR	95% CI	*p*
Age (per year increase)	1·04	1·03 to 1·05	< 0·01	1·05	1·02 to 1·07	< 0·01
Gender						
Male	Ref.			Ref.		
Female	1·11	0·81 to 1·51	0·52	0·74	0·37 to 1·47	0·38
Histology						
Well differentiated	Ref.			Ref.		
Moderately differentiated	1·58	0·91 to 2·73	0·10	2·37	0·88 to 6·38	0·09
Poorly differentiated	2·21	1·22 to 3·98	< 0·01	3·31	1·13 to 9·72	0·03
P 16 Status						
P 16 negative	Ref.			Ref.		
P 16 positive	0·43	0·26 to 0·72	< 0·01	0·50	0·29 to 0·87	0·01
Localization						
Oral cavity/Lips		Ref.			Ref.	
Oropharynx	1·30	0·92 to 1·84	0·14	0·68	0·29 to 1·57	0·36
Larynx	1·20	0·62 to 2·34	0·58	0·60	0·07 to 5·5	0·65
Hypopharynx	1·98	1·37 to 2·87	< 0·01	0·52	0·23 to 1·19	0·12
AJCC Stage						
Stage I	Ref.			Ref.		
Stage II	1·91	0·88 to 4·14	0·10	4·80	1·21 to 19·02	0·03
Stage III	1·40	0·65 to 2·98	0·39	1·90	0·52 to 7·06	0·33
Stage IV	3·68	1·94 to 7·0	< 0·01	6·84	1·95 to 23·97	<0·01
Smoking						
No	Ref.			Ref.		
Yes	1·62	1·13 to 2·33	< 0·01	0·74	0·34 to 1·67	0·48
Ex-Smoker	1·83	1·3 to 2·57	< 0·01	1·15	0·57 to 2·33	0·69
Drinking						
No	Ref.			Ref.		
Yes	1·26	0·86 to 1·85	0·23	2·06	0·88 to 4·83	0·10
Ex-Drinker	1·82	1·26 to 2·62	< 0·01	1·43	0·72 to 2·82	0·31
IHC panel						
0 and 1 genes dysregulated	Ref.			Ref.		
2 and 3 genes dysregulated	1·70	1·27 to 2·27	< 0·01	2·23	1·41 to 3·54	< 0·01

Abbreviations: HR, hazard ratio; Ref, reference.

## DISCUSSION

The major challenge of the post-genomic era is the rational incorporation of the myriad ‘omics’ data points with known clinico-pathologic characteristics, into a complete, reproducible and universal staging system [22]. With the rapid advancement of molecular genomics, there is an enormous resource of publicly available molecular profiles of tumors of different subtypes. The formation of highly organized consortiums aimed at creating a database of molecular profiles matched with clinical data such as the International Cancer Genome Consortium (ICGC) and The Cancer Genome Atlas (TCGA), certainly provide a huge opportunity to explore these possibilities [15, 23]. For HNSCC, like most lethal cancers, reducing mortality requires a combination of more accurate prognostication, development of effective treatment protocols as well as identification of novel therapeutic targets. Refining staging with molecular biomarkers could prognosticate and identify previously unidentified subgroups that require treatment strategies that may be different from conventional staging. When incorporating these biomarkers, it is important to note the basic tenets of staging: it has to be relatively simple, reproducible and universal across different populations. Therefore, the combination of clinical features and well-validated prognostic markers would stratify patients into clinically important clinical subgroups, and the molecular markers should be independent of clinical features. An added bonus would be if these markers could also account for the biology for these tumors instead of being purely molecular passengers.

Using these principles, we designed this study to identify an independently prognostic IHC signature that can be readily applied and reproducible in the clinical setting and used in conjunction with current staging modalities. The derivation of the molecular biomarkers described here was based on publically available TCGA databases for three different tumor subtypes: HNSCC, lung and breast cancer. Using integrative analysis of copy number profiles and gene expression of more than 1366 cancer samples, we identified six putative prognostic targets. This “promiscuous” approach of identifying prognostic targets from three different tumor subtypes not known to have any clinical correlate is deliberate; a small list of molecular targets consistently prognostic in all three different tumor types lends weight to its biological significance and is less likely to be an artifact of genomic profiling. This approach also provides the additional benefit of identifying molecular prognostic targets not specifically limited to one tumor type, but instead underpins the biological basis of multiple types of tumors. Consequently, if any of these markers prove to have a therapeutic window, these could also be applied across multiple tumors through a ‘basket trial’ strategy [24].

In order to validate the strength of these biomarkers, validation studies were performed in an epidemiologically distinct population from TCGA: a large cohort of patients with HNSCC across two high volume centers in Singapore. The differences between the two cohorts conveniently add weight to the observations here, as they demonstrate the validity of the panel described here as being robust enough to be applied even across populations. Ultimately, the data here shows that the gene signature described here, is validated across more than 500 patients with head and neck cancer, regardless of geographical distribution. One important requirement in this analysis was the ability to perform multivariate Cox regression analysis to differentiate the interactions between the putative biomarkers with various known prognostic factors. We have also chosen overall survival as our endpoint as it is more robust and less subjective than recurrence free survival as an endpoint. In addition, despite the whole range of operations performed for tumours in different sites of the head and neck region, our immunohistochemistry panel accurately prognosticates patients highlighting the robustness of the biological significance of the chosen biomarkers.

All three molecular prognostic targets identified from this study have interesting biological roles. ATP13A3 is a member of the P-type ATPase family of proteins that are involved in the transport of a variety of cations across membranes. Recent studies have suggested its overexpression in cervical squamous cell carcinoma versus normal found in focal aberrations showed altered expression concurrent with chromosomal aberration [25]. Work from our laboratory has shown that overexpression of ATP13A3 is associated with upregulation of aurora kinase activity (manuscript in preparation). Potentially, ATP13A3 can serve as a biomarker of susceptibility to inhibition of aurora kinase. ANO1 is a calcium-activated chloride channel (CaCC), which plays a role in transepithelial anion transport and smooth muscle contraction. Interestingly, it has been found to have enhanced expression in HNSCC and strongly correlated with the ability of the HNSCC cells to regulate cell motility and cell migration as well as poor prognosis and overall survival [26, 27]. Finally, SSR3 encodes the gamma subunit of the signal sequence receptor which is a glycosylated endoplasmic reticulum membrane receptor associated with protein translocation across the ER membrane. SSR3 was also identified as a novel candidate oncogene that showed different amplification patterns among different genes within 3q25·3-qter in esophageal squamous cell carcinoma [28].

The power of these biomarkers is the potential for easy adoption and reproducibility. The advent of immunohistochemistry auto-stainers meant that this three gene molecular prognostic panel can be readily adopted by any center worldwide as assay conditions are standardized. Samples can also be processed and interpreted as part of the routine histopathologic analysis and additional processing is not required. With the development of artificial intelligence in interpreting test results, it is conceivable that the IHC interpretation can be done by an automated process and this can provide more uniformity in the interpretation of results and take away subjectivity. Importantly, the IHC panel could potentially be applied to preoperative biopsies for prognostication in patients to further determine the therapeutic approach, thus potentially circumventing problems arising from the use of other techniques where the availability of sufficient tissue for extraction of DNA, RNA and proteins can be challenging.

A major limitation in this study is the possibility that putative prognostic targets from three different tumor types might lead one to conclude that these targets could be identified by “chance events”. It must be noted that with the advent of next generation sequencing, large amounts of mutational data are now deposited in online portals hosted by the International Cancer Genome Consortium (ICGC) and The Cancer Genome Atlas (TCGA) and it would be interesting to see if mutational data can be derived from mining this rich repository of genomic data. If successful, this could provide a sensitive method of detecting prognostic markers via polymerase chain reaction. However, despite the discrepancy in the clinical-pathologic characteristics of patients with HNSCC in the TCGA generation cohort and Singapore validation cohort, three out of four targets which were successfully optimized for IHC proved to be prognostic; further highlighting the fact that the identified prognostic markers are true biological prognostic markers rather than surrogate biomarkers of disease severity and patient characteristics. It would be interesting to decipher if EXOC3, which was not validated to be prognostic, could provide some insight into different tumor biology of HNSCC in a Caucasian versus an Asian population. In addition, given that our scoring was performed on tissue microarrays of HNSCC, it would be pertinent to develop a scoring system for IHC performed on whole tumor slides. This would be important for future studies. In addition, this prognostic panel should be validated by other research groups so as to demonstrate the robustness of the panel across different populations and staining technologies.

In conclusion, our study shows that a three gene molecular prognostic panel can provide prognostic information in patients with HNSCC independently of clinical prognosis variables. These data supports the theory that distinct molecular features govern the clinical phenotypes of this disease. This panel can be used as an adjunct to current staging systems to provide pertinent prognostic information. In addition, we have demonstrated that important molecular and clinically relevant information could be identified from the huge archive of molecular profiles of various tumor types that is now publicly available. This valuable resource should be mined extensively to improve knowledge of tumor biology and improve patient care in the long term.

## MATERIALS AND METHODS

### Identification of potential prognostic markers

To identify clinically relevant prognostic markers, we performed integrative analysis on gene expression and copy number data, and determined their relationship to clinical outcomes. SNP6 data for copy number aberrations, and RNASeq for gene expression data for HNSCC (*n =* 276), breast (*n =* 808) and lung cancers (*n =* 282) (Supplementary Figure 3) were obtained from TCGA (syn2812925) [29]. We used the level 3 processed data provided by TCGA, representing normalized copy number ratios (SNP6 data) or normalized gene read counts (RNASeq data). Genes present in the region of focal copy number gains as reported by the TCGA GISTIC analysis in HNSCC were assessed for an association between gene expression and survival using the Gehan-Breslow-Wilcoxon test in all three datasets. No multiple testing corrections were applied to the discovery set to maximize gene recall for validation.

The algorithm for filtering the genes (Supplementary Table 4) is as follows:

1. Presence in the same chromosomal arm of a GISTIC amplicon
2. Prognostic by copy number OR expression in the HNSCC cohort (*p <* 0.05) and in either:
  a. Lung Squamous Cell Carcinoma (LUSC) (*p <* 0.05)
  b. Breast Invasive Carcinoma (BRCA) (*p <* 0.05).
3. Frequent copy number gain in the HSNC cohort (> 5%) and in either:
  a. Lung Squamous Cell Carcinoma (LUSC) (> 5%).
  b. Breast Invasive Carcinoma (BRCA) (> 5%).
4. Infrequent copy number loss in all datasets (< 2%).

This gave a final list of 85 genes (Supplementary Table 5) which were manually inspected to select the final 6.

### Study population

The selected genes were validated on a cohort of 333 patients treated for histologically confirmed HNSCC and who underwent treatment at the National Cancer Centre Singapore or the Singapore General Hospital between 1998 and 2010. Treatment decisions were based on a multi-disciplinary tumor board. All patients underwent surgery with or without adjuvant treatment (adjuvant radiotherapy and/or chemotherapy). This study was approved by the Singhealth Centralized Institutional Review Boards (CIRB 2011/678/B and 2007/438/B). A consecutive series of patients were included based on the availability of clinic-pathologic, treatment and outcome data, and access to sufficient archived tissue samples to construct a series of tissue microarrays (TMA). The clinical characteristics of this cohort as well as the HNSCC cohort of the TCGA dataset with available clinical information are listed in Table 2. Tissue microarrays were constructed as previously described [30]. Due to loss of TMA cores during the immunohistochemical staining process, 96.7% of all the cores were retained and considered in the staining analysis.

**Table 2:** Demographic and clinical characteristics of patients of the examined cohorts

Characteristics	TCGA		Singapore		*p*
	(*n =* 200)		(*n =* 333)		
	No.	%	No.	%	
Age at diagnosis, years					0·63
Mean	61·6		61·9		
Rnge	19–90		27–92		
Gender					< 0·01
Male	140	73·3	101	30·5	
Female	51	26·7	230	69·5	
Follow-up time, months					< 0·01
Mean	25·71		39·69		
Range	1–137		1–186		
Primary Site					< 0·01
Oral cavity/Lips	131	65·8	131	39·3	
Oropharynx	14	7	107	32·1	
Larynx	52	26·1	14	4·2	
Hypopharynx	2	1	68	20·4	
Maxillary Sinus	0	0	9	2·7	
Nasopharynx	0	0	4	1·2	
Histology grade (differentiation)					< 0·01
Well	13	6·5	34	12	
Moderate	130	65	161	56·9	
Poor	54	27	65	23	
Unknown	3	1.5	23	8·1	
Pathologic T stage					< 0·01
T1	9	4.5	57	37·8	
T2	57	28·6	95	16·5	
T3	64	32·2	55	28·5	
T4	69	34·7	126	17·1	
Pathologic N stage					0·32
N0	84	43·3	125	37·5	
N1	28	14·4	59	17·7	
N2	78	40·2	135	40·5	
N3	4	2·1	14	4·2	
Pathologic M Stage					0·41
M0	196	98·5	322	96·7	
M1	3	1·5	10	3	
AJCC Stage					< 0·01
Stage I	8	4·1	32	9·6	
Stage II	53	27·5	35	10·5	
Stage III	39	20·2	54	16·2	
Stage IV	93	48·2	212	63·7	
Smoking					< 0·01
Active smoker	73	36·5	104	15	
Ex-smoker	81	40·5	75	22·5	
Never smoker	38	19	139	41·7	
Alcohol					< 0·01
Active drinker	131	65·5	55	16·5	
Ex drinker			62	18·6	
Never drinker	64	32	173	52	
Satus					< 0·01
Alive	107	56	133	39·9	
Dead	84	44	196	58·9	

Although patients in both cohorts were diagnosed with the same histological condition, there are noticeable differences in the epidemiology of the two cohorts (Table 2). As previously reported, there were significantly more female patients in the Singapore cohort versus the TCGA cohort (69·5% vs 26·7% respectively, *p <* 0·01) [31]. A number of lifestyle-dependency factors were different between the cohorts. There was a higher proportion of previous or active smokers in the TCGA cohort (77%) compared to the Singapore cohort (37·5%; *p <* 0·01). A similar difference can be found for the consumption of alcohol (*p <* 0·01). In addition, there were also differences in the distribution of the primary tumor sites, although this may be due to issues relating to specimen and tissue availability rather than true epidemiological differences. In our cohort, P16 status was only tested in patients with hypopharyngeal and oropharyngeal tumours (52.5%).

### Immunohistochemistry

**Figure 4:**
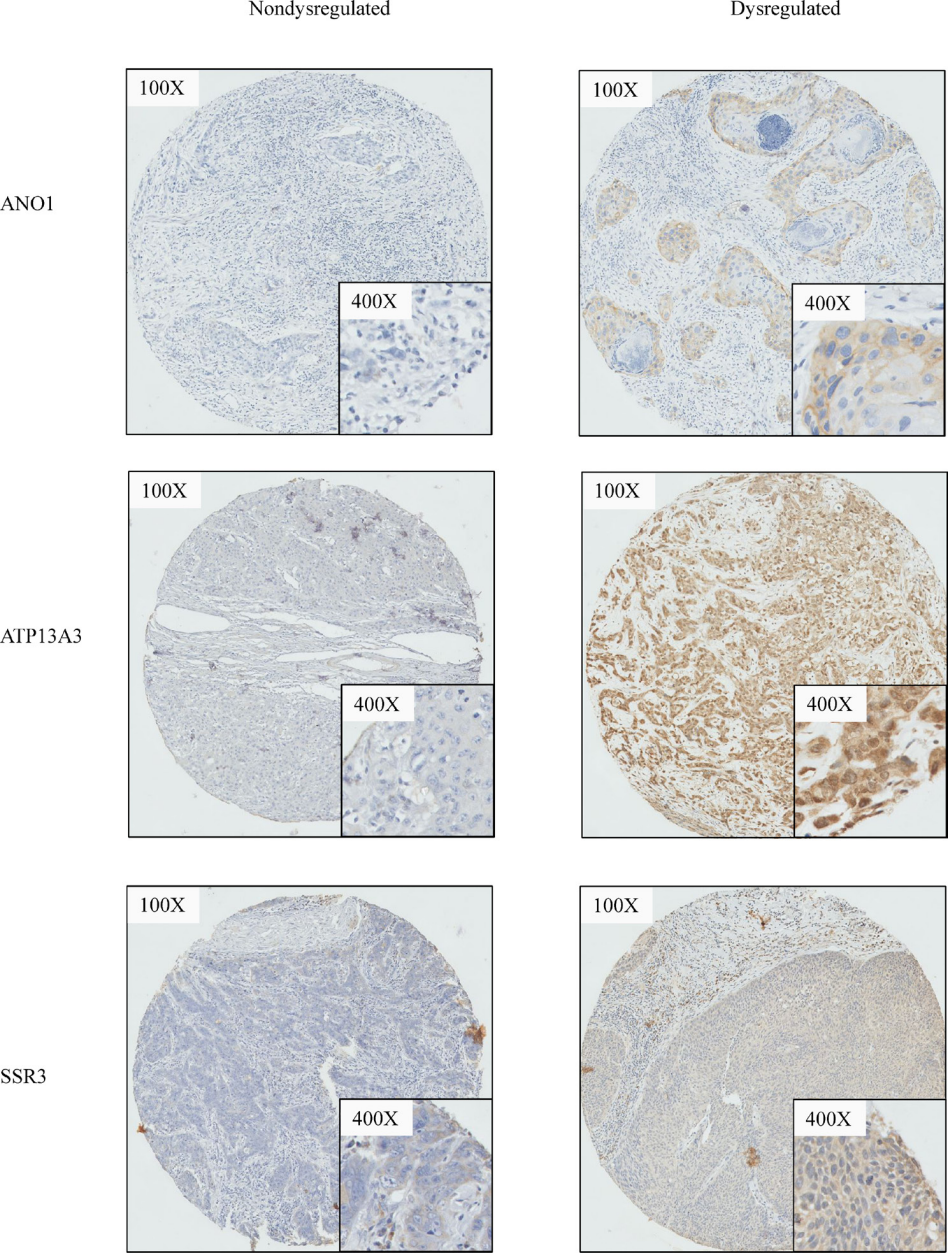
Representative cases illustrating immunohistochemistry staining For ANO1, ATP13A3, SSR3, representative cases which demonstrates dysregulation versus non-dysregulation of the respective biomarker are shown.

All IHC was performed using the Bond System (Leica Microsystems, Ltd, Milton Keynes, UK) according to the manufacturer’s recommendations. Antibody sources and conditions used for IHC are detailed in Supplementary Table 6. Of the six targets selected, only 4 targets were successfully optimized for immunohistochemistry, and staining performed on the TMAs above. The staining of each core on the TMA was scored from 0 to 3 (Supplementary Figure 4) by two independent researchers blinded to the outcome (SM and LSM). Samples were then characterized as having overexpression (score, 2–3) or underexpression (score, 0–1) of the target. Where there was discrepancy in the scores, a third scorer (CAJ) scored the cores independently to determine the assigned expression level of each target (Figure 4).

### Clinical endpoints and statistical analysis

Primary endpoint of the study was overall survival (OS), with death of any cause defined as the endpoint. Kaplan-Meier curves were plotted to compare OS depending on the number of dysregulated genes. Student’s *t*-test or Mann-Whitney *U* test was used to compare continuous demographic and clinical factors between the two cohorts, whereas chi–square was used for categorical variables. Cox univariate regression was used to determine the HR of individual clinical factors and genes included in the prognostic panel. Cox proportional hazard model was employed to test the independent association of the prognostic gene panel with OS after adjusting for clinical and demographic factors. All statistical analysis was done using SPSS (Version 18, IBM Armonk, NY). A *p*-value of less than 0·05 was considered statistically significant.

## SUPPLEMENTARY MATERIALS FIGURES AND TABLES




