# Supplementary material for: A three gene immunohistochemical panel serves as an adjunct to clinical staging of patients with head and neck cancer
Source: Oncotarget. 2017 Jun 19;8(45):79556–66. doi: 10.18632/oncotarget.18568 (PMC5668068; doi:10.18632/oncotarget.18568)
Supplement: Supplementary file 1 [file oncotarget-08-79556-s001.pdf]

# A three gene immunohistochemical panel serves as an adjunct to clinical staging of patients with head and neck cancer

## Supplementary Materials

**Supplementary Table 1: Individual HR of each investigated prognostic marker in a univariate and multivariate analysis**

| Markers | HR | 95% CI | *p* |
|---|---|---|---|
| ANO1 | | | |
| not overexpressed | Ref. | | |
| overexpressed | 1·72 | 1·21 to 2·46 | 0·003 |
| ATP13A3 | | | |
| not overexpressed | Ref. | | |
| overexpressed | 1·45 | 1·09 to 1·93 | 0·011 |
| SSR3 | | | |
| not overexpressed | Ref. | | |
| overexpressed | 1·82 | 1·20 to 2·78 | 0·005 |
| EXOC3 | | | |
| not overexpressed | Ref. | | |
| overexpressed | 1·12 | 0·84 to 1·49 | 0·438 |

Abbreviations: ANO1, Anoctamin-1. ATP13A3, ATPase Type 13A3. SSR3, Signal Sequence Receptor 3. EXOC3, Exocyst Complex Component 3. Ref, reference.

**Supplementary Table 2: Number of dysregulated markers per AJCC stage in the validation cohort**

| No. of dysregulated molecular markers | *n* | % (Stage) | % Total |
|---|---|---|---|
| Stage I | | | |
| 0 | 9 | 31 | 2·8 |
| 1 | 7 | 24·1 | 2·2 |
| 2 | 12 | 41·4 | 3·7 |
| 3 | 1 | 3·4 | 0·3 |
| Stage II | | | |
| 0 | 8 | 23·5 | 2·5 |
| 1 | 10 | 29·4 | 3·1 |
| 2 | 13 | 38·2 | 4 |
| 3 | 3 | 8·8 | 0·9 |
| Stage III | | | |
| 0 | 6 | 11·5 | 1·9 |
| 1 | 22 | 42·3 | 6·8 |
| 2 | 19 | 36·5 | 5·9 |
| 3 | 5 | 9·6 | 1·6 |
| Stage IV | | | |
| 0 | 20 | 9·7 | 6·2 |
| 1 | 77 | 37·2 | 23·9 |
| 2 | 91 | 44 | 28·3 |
| 3 | 19 | 9·2 | 5·9 |

**Supplementary Table 3: Post-hoc power calculation of targets ($\alpha = 0·05$)**

| Gene | Power |
|---|---|
| ATP13A3 | 0·92 |
| SSR3 | 0·99 |
| ANO1 | 0·94 |
| EXOC3 | 0·18 |
| Combined panel (0/1 vs 2/3) | 1 |

**Supplementary Table 4: Algorithm to derive the prognostic targets**

| Filtering steps | Number of genes remaining at each stage |
|---|---|
| Initial number of genes assessed | 15911 |
| Genes in same arm as GISTIC amplicon | 7704 |
| Genes prognostic by copy number or expression in HNSCC < 0.05 | 1995 |
| Same in lung or breast | 1190 |
| Copy number gain in HNSCC > 5% | 174 |
| Copy number gain in lung/breast > 5% | 160 |
| Copy number loss in any dataset < 2% | 85 |
| Manual inspection of 85 genes | 6 |

**Supplementary Table 5: List of 85 genes before manual inspection to shortlist the final 6 targets.**
See Supplementary_Table_5

**Supplementary Table 6: Sources for antibodies used in the study and the conditions used for optimum staining**

| Target | Product number | Antibody source | BOND Immunohistochemistry conditions |
|---|---|---|---|
| ANO1 | LS-C88846-1 | LifeSpan Biosciences, Inc, Seattle, WA | ER1, Bond Refine kit, Ab 1:200, 20 mins at 25 Deg C |
| ATP13A3 | LS-C156640 | LifeSpan Biosciences, Inc, Seattle, WA | ER1, Bond Refine kit, Ab 1:150, 20 mins at 25 Deg C |
| SSR3 | ab121458 | Abcam Plc, Cambridge, UK | ER1, Bond Refine kit, Ab1:250, 20 mins at 25 Deg C |
| EXOC3 | LS-B2988-50 | LifeSpan Biosciences, Inc, Seattle, WA | ER1, Bond Refine Kit, Ab 1:1000, 20 mins at 25 Deg C |

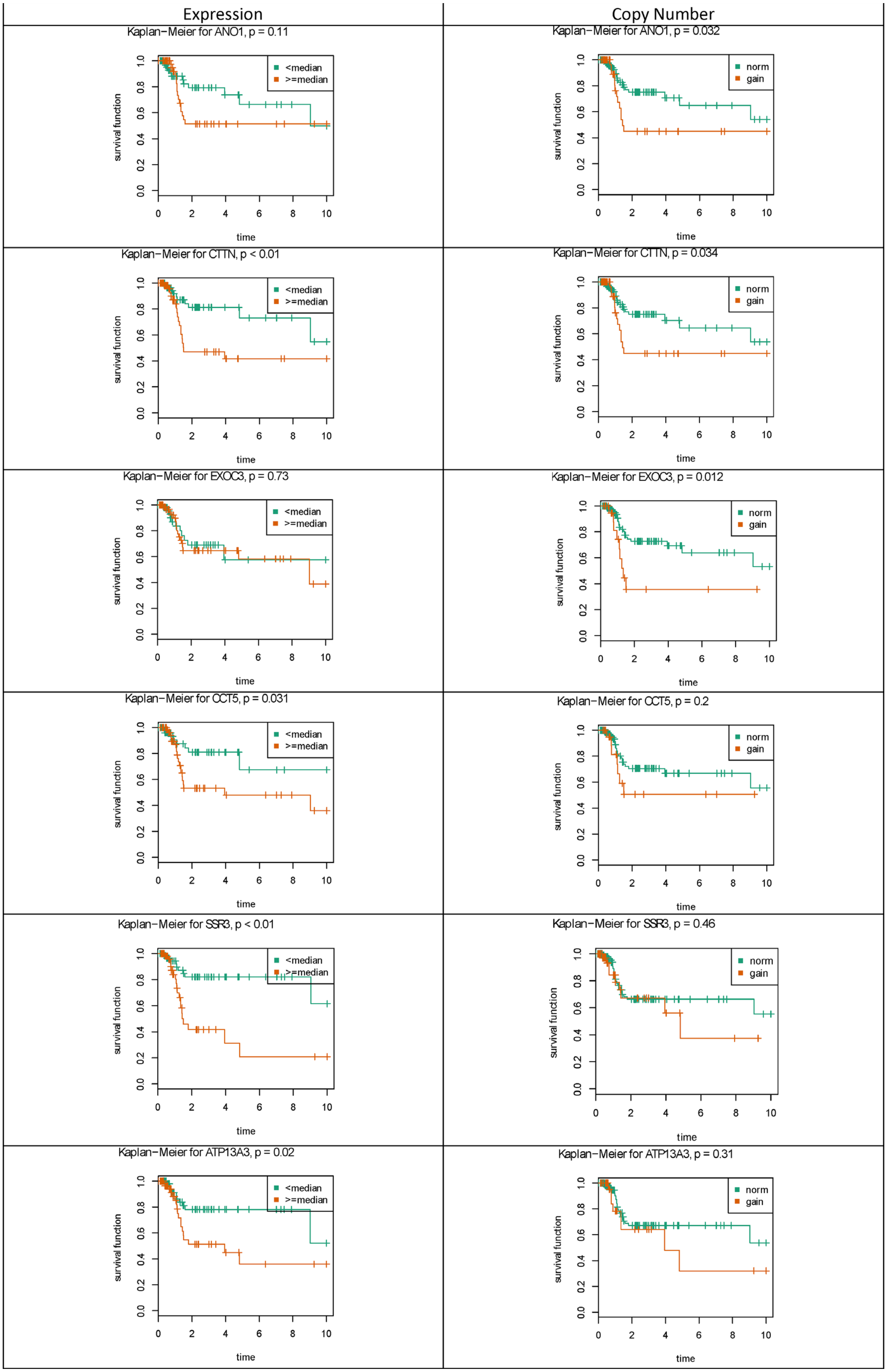


**Supplementary Figure 1: Kaplan-Meier curves for selected targets.**

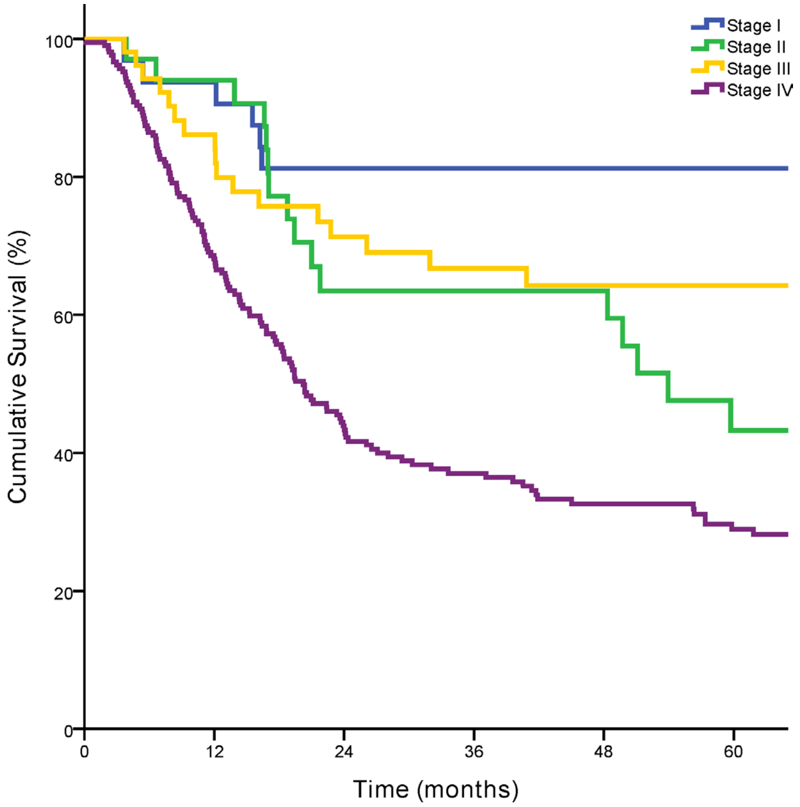


No. at risk

| | | | | | | |
|---|---|---|---|---|---|---|
| Stage I | 32 | 30 | 24 | 17 | 15 | 15 |
| Stage II | 35 | 28 | 18 | 18 | 16 | 10 |
| Stage III | 54 | 42 | 32 | 28 | 26 | 25 |
| Stage IV | 211 | 135 | 80 | 60 | 48 | 39 |

**Supplementary Figure 2: Kaplan-Meier estimate of survival per AJCC stage in the Singapore cohort ($p < 0·01$).**

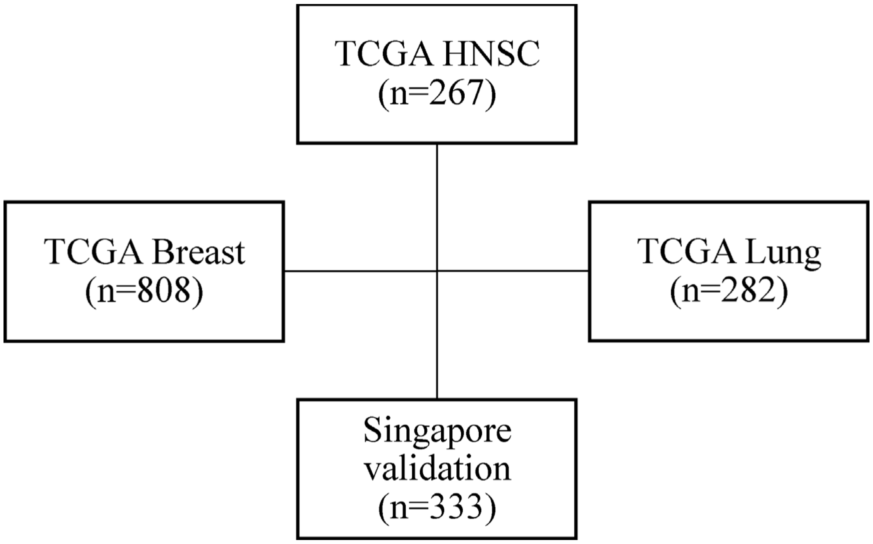


**Supplementary Figure 3: Flow-chart diagram illustrating the information flow of extracted and analysed sub-sets of patients.**

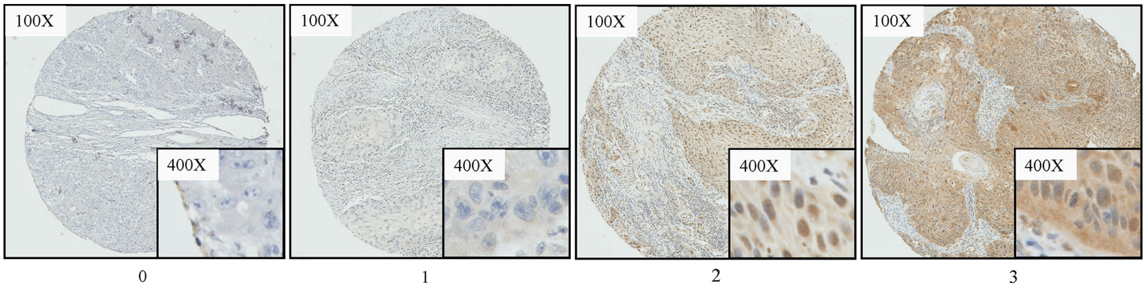


**Supplementary Figure 4: Representative images demonstrating the scores 0 to 3 for tumour scoring.**
