# Supplementary material for: A three gene immunohistochemical panel serves as an adjunct to clinical staging of patients with head and neck cancer
Source: Oncotarget. 2017 Jun 19;8(45):79556–66. doi: 10.18632/oncotarget.18568 (PMC5668068; doi:10.18632/oncotarget.18568)
Supplement: Supplementary file 2 [file oncotarget-08-79556-s002.docx]

| Supplementary table 5: List of 85 genes before manual inspection to shortlist the final 6 targets. | | |
| --- | --- | --- |
| Gene Symbol | Gene IDs | Cytoband |
| CTHRC1 | 115908 | 8q22.3 |
| LPCAT1 | 79888 | 5p15.33 |
| FBXO45 | 200933 | 3q29 |
| ATP13A3 | 79572 | 3q29 |
| EIF5A2 | 56648 | 3q26.2 |
| DERL1 | 79139 | 8q24.13 |
| SKP2 | 6502 | 5p13 |
| WDR53 | 348793 | 3q29 |
| SQLE | 6713 | 8q24.1 |
| TP53INP2 | 58476 | 20q11.22 |
| TM4SF19 | 116211 | 3q29 |
| PLEKHG4B | 153478 | 5p15.33 |
| PSCA | 8000 | 8q24.2 |
| CA3 | 761 | 8q21.2 |
| TTPAL | 79183 | 20q13.12 |
| ANO1 | 55107 | 11q13.3 |
| PLOD2 | 5352 | 3q24 |
| GOLIM4 | 27333 | 3q26.2 |
| YWHAZ | 7534 | 8q23.1 |
| UBXN7 | 26043 | 3q29 |
| FYTTD1 | 84248 | 3q29 |
| IL7R | 3575 | 5p13 |
| CCT5 | 22948 | 5p15.2 |
| C5orf51 | 285636 | 5p13.1 |
| SSR3 | 6747 | 3q25.31 |
| RPL39L | 116832 | 3q27 |
| PPFIA1 | 8500 | 11q13.3 |
| DHCR7 | 1717 | 11q13.4 |
| SENP2 | 59343 | 3q27.2 |
| GPR87 | 53836 | 3q24 |
| RNF168 | 165918 | 3q29 |
| SLC33A1 | 9197 | 3q25.31 |
| PDCD10 | 11235 | 3q26.1 |
| PRKCI | 5584 | 3q26.3 |
| IL1RAP | 3556 | 3q28 |
| OSR2 | 116039 | 8q22.2 |
| LMLN | 89782 | 3q29 |
| GAPDH | 2597 | 12p13 |
| KNG1 | 3827 | 3q27 |
| DGKG | 1608 | 3q27.2-q27.3 |
| FAM105B | 90268 | 5p15.2 |
| HSF1 | 3297 | 8q24.3 |
| CLDND1 | 56650 | 3q12.1 |
| GNB4 | 59345 | 3q26.33 |
| MRPL47 | 57129 | 3q26.33 |
| TRHR | 7201 | 8q23 |
| SLC12A7 | 10723 | 5p15 |
| RYK | 6259 | 3q22 |
| KIAA0226 | 9711 | 3q29 |
| C1RL | 51279 | 12p13.31 |
| TPPP | 11076 | 5p15.3 |
| KLHL38 | 340359 | 8q24.13 |
| CTTN | 2017 | 11q13 |
| P2RY12 | 64805 | 3q24-q25 |
| RPL8 | 6132 | 8q24.3 |
| ATP11B | 23200 | 3q27 |
| UGT3A2 | 167127 | 5p13.2 |
| RASA2 | 5922 | 3q22-q23 |
| ZDHHC23 | 254887 | 3q13.31 |
| ZNF16 | 7564 | 8q24 |
| FBXO4 | 26272 | 5p12 |
| TM4SF4 | 7104 | 3q25 |
| ACAP2 | 23527 | 3q29 |
| MCF2L2 | 23101 | 3q27.1 |
| NIT2 | 56954 | 3q12.2 |
| AGTR1 | 185 | 3q24 |
| SLC2A2 | 6514 | 3q26.1-q26.2 |
| RPL22L1 | 200916 | 3q26.2 |
| ODF1 | 4956 | 8q22.3 |
| NADSYN1 | 55191 | 11q13.4 |
| PLCH1 | 23007 | 3q25.31 |
| SHANK2 | 22941 | 11q13.2 |
| GYG1 | 2992 | 3q24-q25.1 |
| PDCD6 | 10016 | 5p15.33 |
| TRPC1 | 7220 | 3q23 |
| GPR171 | 29909 | 3q25.1 |
| THEM6 | 51337 | 8q24.3 |
| EXOC3 | 11336 | 5p15.33 |
| PRLR | 5618 | 5p13.2 |
| CYCS | 54205 | 7p15.3 |
| FBXO32 | 114907 | 8q24.13 |
| B3GALNT1 | 8706 | 3q25 |
| PEX5L | 51555 | 3q26.33 |
| P2RY14 | 9934 | 3q24-q25.1 |
| RDH10 | 157506 | 8q21.11 |
